# Development and Evaluation of Microemulsions for Transdermal Delivery of Insulin

**DOI:** 10.5402/2011/780150

**Published:** 2011-07-07

**Authors:** Jadupati Malakar, Suma Oomen Sen, Amit Kumar Nayak, Kalyan Kumar Sen

**Affiliations:** ^1^Department of Pharmaceutics, Bengal College of Pharmaceutical Sciences and Research, West Bengal, Durgapur 713212, India; ^2^Department of Pharmaceutics, Gupta College of Technological Sciences, West Bengal, Asansol 713301, India; ^3^Department of Pharmaceutics, Seemanta Institute of Pharmaceutical Sciences, Orissa, Mayurbhanj 757086, India

## Abstract

Insulin-loaded microemulsions for transdermal delivery were developed using isopropyl myristate or oleic acid as the oil phase, Tween 80 as the surfactant, and isopropyl alcohol as the cosurfactant. The pseudoternary phase diagrams were constructed to determine the composition of microemulsions. The insulin permeation flux of microemulsions containing oleic acid as oil phase through excised mouse skin and goat skin was comparatively greater than that of microemulsions containing isopropyl myristate as oil phase. The insulin-loaded microemulsion containing 10% oleic acid, 38% aqueous phase, and 50% surfactant phase with 2% dimethyl sulfoxide (DMSO) as permeation enhancer showed maximum permeation flux (4.93 ± 0.12 *μ*g/cm^2^/hour) through goat skin. The *in vitro* insulin permeation from these microemulsions was found to follow the Korsmeyer-Peppas model (R^2^ = 0.923 to 0.973) over a period of 24 hours with non-Fickian, “anomalous” mechanism. Together these preliminary data indicate the promise of microemulsions for transdermal delivery of insulin.

## 1. Introduction

Microemulsion is defined as a dispersed system consisting of oil, surfactant, cosurfactant, and an aqueous phase at appropriate ratios [1]. It is a thermodynamically stable optically transparent isotropic liquid solution with a droplet diameter usually less than 100 nm [2]. Unlike coarse emulsions micronized with external energy microemulsions are based on low interfacial tension. This is achieved by adding a cosurfactant, which leads to spontaneous formation of a thermodynamically stable microemulsion. Several interesting characteristics of microemulsions, namely, enhanced drug solubilization, good thermodynamic stability, ease of preparation, low viscosity, high drug loading capacity, and small droplet size, have drawn attention for their use as vehicles for drug delivery [3–5]. Although microemulsions can be used to deliver drugs via several routes, these versatile systems have been extensively studied as vehicles for transdermal administration and have attracted much attention in recent years [1, 4, 6–9]. As vehicle for transdermal systems, microemulsions can increase the local or systemic delivery of drugs by different mechanisms [3]. First, their composition and structure enable them to incorporate a greater amount of drug than other conventional topical formulations such as ointments, creams, gels, and lotions. Second, the diffusional barrier of the skin may be modified depending on the composition of the microemulsion. Third, an increased thermodynamic activity of the drug may favour its partitioning into the skin.

The first protein used to treat disease was insulin that revolutionized the diabetic treatment [10, 11]. Insulin was widely used clinically for the treatment of insulin-dependent diabetes mellitus (IDDM) or type-I diabetes [12]. Insulin is a peptide hormone composed of 51 amino acid residues and has molecular weight about 5.7 kDa [13]. The molecular structure of insulin is composed of 2 peptide chains: A chain (21 amino acid residues) and B chain (30 amino acid residues) [14]. Conventional insulin treatment is basically a replacement therapy, in which exogenous insulin is administered subcutaneously to mimic, as closely as possible, the insulin secretion of the healthy pancreas. The subcutaneous route has been the mainstay of insulin delivery until now. Although parenteral insulin is satisfactory in terms of efficacy in the great majority of cases, this is associated with some serious adverse effects like peripheral hyperinsulinemia, the stimulation of smooth muscle cell proliferation, and the incorporation of glucose into the lipid of arterial walls, and they might therefore be the causative factor in diabetic micro- and macroangiopathy [15]. In addition, the burden of daily injections, physiological stress, pain, inconvenience, cost, infection, inability to handle insulin, and the localized deposition of insulin leads to a local hypertrophy and fat deposition in the injection sites [14, 16]. To limit these shortcomings, the delivery of insulin by nonparenteral routes has gained significant attention over the last 2 decades. Among them, transdermal route for insulin delivery as an alternative nonparenteral route of administration offers the advantage in terms of patient compliance and can be used to treat diabetic patients. During the last few years, various experimental methodologies have been successfully developed for facilitating transdermal delivery of insulin [15, 17–22]. 

In this present investigation, the transdermal delivery system of insulin through microemulsions was investigated. Insulin-loaded microemulsions for transdermal delivery, containing isopropyl myristate or oleic acid as the oil phase, Tween 80 as the surfactant, isopropyl alcohol as the cosurfactant, were prepared. We have selected these components for the formulation of insulin-loaded microemulsions due to their skin permeation capacity [1]. The objective of the present study is to produce a safe and painless insulin delivery system and determine the permeation flux of different microemulsion formulations through the skin. We also have studied the transdermal permeation of insulin from the best formulations with adding 2% dimethyl sulfoxide (DMSO) as an additional permeation enhancer for further insulin permeation improvement. 

## 2. Materials and Methods

### 2.1. Materials

Human insulin, 40 IU/mL (Torrent Pharmaceutical Ltd., India), oleic acid (Fine Chemicals Ltd., India), isopropyl alcohol (Qualigens Fine Chemicals, India), isopropyl myristate (Loba Chemie Pvt. Ltd., India), Tween 80, and dimethyl sulfoxide (Merck Specialties Pvt. Ltd., India) were used. All chemicals and reagents used were of analytical grade.

### 2.2. Construction of Pseudoternary Phase Diagram

Microemulsions were prepared by using conventional titration method. The oil (isopropyl myristate or oleic acid) and aqueous phases were first combined with the surfactant (Tween 80). Cosurfactant (isopropyl alcohol) was added gradually with magnetic stirring at room temperature until the system was transparent. Transparent, single-phase formulations were indicative of stable microemulsions. Microemulsions were allowed to equilibrate with gentle magnetic stirring for 15 minutes. 

These microemulsions were then titrated with water using a micropipette at room temperature. Then, these were stirred vigorously for a sufficient length of time and end point (onset of turbidity or phase separation) was visually monitored against a dark background by illuminating the samples with a white light. The experiments were performed in triplicate to check reproducibility. From the end point composition of titrated samples, the mass percent composition of the components like oil, surfactant, and water was calculated and plotted on triangular coordinates to construct pseudoternary phase diagrams [23]. From the microemulsion regions in the pseudoternary phase diagram, the three different formulas for the development microemulsions were selected and prepared. The compositions of selected microemulsions are shown in Table 1.

### 2.3. Preparation of Insulin-Loaded Microemulsions

Insulin-loaded microemulsions were prepared by dissolving insulin in water (1.4 mg/mL, i.e., 40 IU/mL) and poured dropwise to oil (isopropyl myristate or oleic acid) and surfactant (Tween 80)/cosurfactant (Isopropyl alcohol) mixture with vigorous stirring at room temperature using composition of selected formulas (Table 1) until the transparent microemulsions were produced. These insulin-loaded microemulsions were allowed to equilibrate with gentle magnetic stirring for 15 minutes. Then various formulated microemulsions were passed through Whatman filter paper (no. 40).

### 2.4. Droplet Size and Zeta Potential Determination

At first, the microemulsion 0.1 mL was diluted to 10 mL of doubled distilled water, and then droplet size and zeta potential were determined by a laser scattering particle size analyzer (MALVERN ZETASIZER, MAL500999). 

### 2.5. Stability of Microemulsions

Physical stabilities of microemulsions were evaluated by centrifugation (Research centrifuge, Remi Instruments, India) at 1250 rpm for a period of 5 hours and then examined for any phase separation [24].

### 2.6. Preparation of Skin for In Vitro Skin Permeation Study

#### 2.6.1. Mouse Skin

Swiss albino mice weighing 80–100 gm were selected for preliminary permeation study, and the study was conducted with the approval of Institutional Animal Ethical Committee. The mice were sacrificed using anesthetic ether. Then the hair from their abdominal region was removed using an animal hair clipper, and, subsequently, full thickness of the skin was harvested. The fatty layer, adhering to the dermis side, was removed by surgical scalpel.

#### 2.6.2. Goat Skin

Selected formulations were further studied for skin permeation using goat ear skin, obtained from the slaughter house after sacrificing the animal within 1 hour. Then the hair was removed from the upper portion of skin surface using an animal hair clipper, and, subsequently, full thickness of the skin was harvested. The fatty layer, adhering to the dermis side, was removed by surgical scalpel. 

Finally, these excised skins were thoroughly rinsed with distilled water and packed in aluminum foils. The skin samples were stored at −20°C and used within a week. 

### 2.7. In Vitro Skin Permeation Study by Franz Diffusion Cell

*In vitro* skin permeation studies were carried out using Franz diffusion cell. The cell consists of two chambers, the donor and the receptor compartment with a diffusion area of 1.43 cm^2^. The donor compartment was open at the top and was exposed to atmosphere. The excised mouse skin was mounted between the compartments of the diffusion cell with stratum corneum facing the donor compartment and clamped into position. Magnetic stirrer bars were added to the receptor chambers and filled with the receptor phase. Phosphate buffer saline (PBS), pH 7.4, was used as receptor medium. The small concentration of sodium azide (0.0025% w/v) was added to prevent any microbial growth [25]. The entire setup was placed over magnetic stirrer, and the temperature was maintained at 37 ± 0.5°C. The skin sections were initially left in the Franz cells for 2 hours in order to facilitate hydration of the skin samples. After this period, 5 ml of the appropriate formulation was applied onto the surface of the skin. 1 mL of medium was collected from receptor compartment at predetermined intervals over study period and replaced with the same amount of fresh buffer. The amount of permeated drug was measured using UV-Visible spectrophotometer (Thermo Spectronic UV-1, USA) by measuring absorbance at *λ*_*Max*⁡_ 214 nm.

### 2.8. Permeation Data Analysis

#### 2.8.1. Kinetics

The data of *in vitro *insulin permeation from various insulin-loaded microemulsions were evaluated kinetically using various mathematical models like zero-order, first-order, Higuchi, and Korsmeyer-Peppas model equations.

Zero-order kinetics: *F* = *K*_*o*_ *t*, where *F* represents the fraction of drug released in time *t* and *K*_*o*_ is the zero-order release constant.
First-order kinetics: ln⁡(1 − *F*) = −*K*_1_ *t*, where *F* represents the fraction of drug released in time *t* and *K*_1_ is the first-order release constant.
Higuchi model: *F* = *K*_*H*_ *t* ^1/2^, where *F* represents the fraction of drug released in time *t* and *K*_*H*_ is the Higuchi dissolution constant.
Korsmeyer-Peppas model: *F* = *K*_*p*_ *t* ^*n*^, where *F* represents the fraction of drug released in time *t* and *K*_*p*_ is the Korsmeyer-Peppas release rate constant, and *n* is the diffusion exponent.

#### 2.8.2. Permeation Flux

The amount of insulin from various insulin-loaded microemulsions was permeated through mice and goat skins were plotted against the function of time. The slope and intercept of the linear portion of plots were derived by regression. The permeation flux was calculated as the slope divided by the skin surface area [26–28]: 

*J*_*ss*_ = (*dQ*/*dt*)_*ss*_ · 1/*A*, where *J*_*ss*_ is the steady-state permeation flux (*μ*g/cm^2^/hour), *A* is the area of skin tissue (cm^2^) through which drug permeation takes place, and (*dQ*/*dt*)_*ss*_ is the amount of drug passing through the skin per unit time at a steady state (*μ*g/hour).

## 3. Results and Discussion

### 3.1. Pseudoternary Phase Diagrams

**Figure 1:**
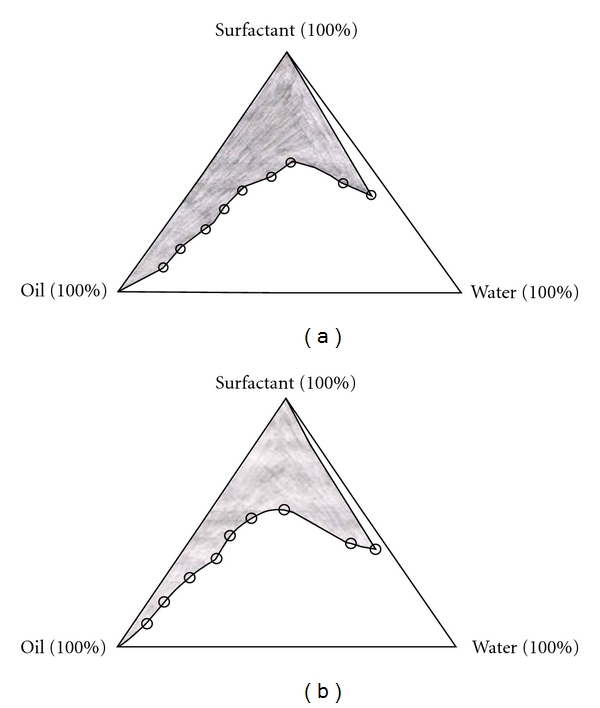
Pseudoternary phase diagrams of (a) oil (isopropyl myristate), surfactant (Tween 80/isopropyl alcohol, in 3 : 1 ratio), and aqueous phases and of (b) oil (oleic acid), surfactant (Tween 80/isopropyl alcohol, in 3 : 1 ratio) and aqueous phases. The shaded area signifies the microemulsion zone.

The pseudoternary phase diagrams of the investigated systems were constructed to determine the composition of an aqueous phase, an oil phase containing isopropyl myristate or oleic acid, and a surfactant/cosurfactant (3 : 1) phase containing Tween 80 as surfactant and isopropyl alcohol as cosurfactant for the formulation of microemulsions (transparent solutions) at room temperature, which were represented in Figures 1(a) and 1(b), as shaded area. From the microemulsion regions in the pseudoternary phase diagrams, six formulas were selected for the development of microemulsions (Table 1, F1 to F6). Using the composition of selected formulas, insulin-loaded microemulsions were formulated and investigated.

### 3.2. In Vitro Skin Permeation

**Figure 2:**
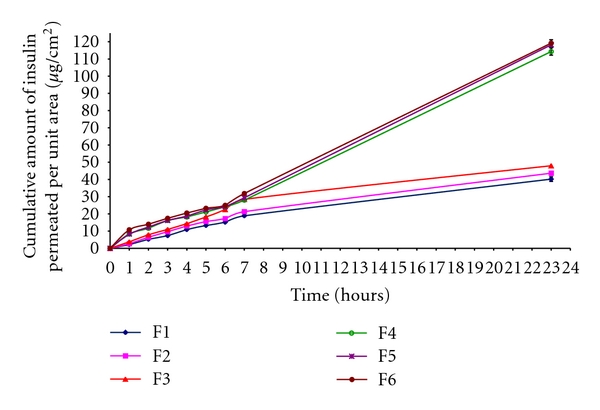
*In vitro* insulin permeation profile through mouse skin per unit area from insulin-loaded microemulsions (mean ± standard error, *n* = 3).

These insulin-loaded microemulsions were studied for *in vitro *skin permeation through excised mouse skin. The amount of insulin permeated through excised mouse skin over 24-hour period was plotted against the function of time (Figure 2), and this result showed higher permeation profile for microemulsion F6 than others over 24 hours. The permeation fluxes (*μ*g/cm^2^/hour) for all these microemulsions through the mouse skin were determined. The determined permeation fluxes are given in Table 1. Among all formulations, the highest permeation flux of 5.09 ± 0.07 *μ*g/cm^2^/hour was observed in case of formulation F6, which contained lower amount of oleic acid (10%) and considerably higher amount of aqueous phase (40%) and surfactant phase (50%). Among microemulsions formulated using isopropyl myristate as oil phase, the higher permeation flux of 2.45 ± 0.05 *μ*g/cm^2^/hour) was observed in case of formulation F3, which contained lower amount of isopropyl myristate (30%) and considerably lower amount of aqueous phase (10%) and surfactant phase (60%). The permeation flux of microemulsions containing oleic acid as oil phase was comparatively greater than that of microemulsions containing isopropyl myristate as oil phase. It is also clear that the insulin permeation was increased with the increase in the amount of surfactant phase and aqueous phase in their composition. This is attributed to skin permeation enhancement capacity by the surfactants. Surfactants can loosen or fluidize the lipid matrix of the stratum corneum—the principal diffusional barrier of the skin—and act as skin permeation enhancer [6]. Also, other components such as isopropyl myristate or oleic acid, which were used as oil phase in these formulated microemulsions, serve as skin permeation enhancers. 

In order to predict and correlate the *in vitro *insulin permeation behavior from these insulin-loaded microemulsions through excised mouse skin, it is necessary to fit into a suitable mathematical model. The *in vitro *insulin permeation data from microemulsions containing insulin through excised mouse skin were evaluated kinetically by various mathematical models like zero-order, first-order, Higuchi, and Korsmeyer-Peppas model. The results of the curve fitting into these above-mentioned mathematical models indicate the *in vitro *insulin permeation behavior of insulin-loaded microemulsions (F1 to F6) (Table 2). When respective correlation coefficients were compared, F1, F2, and F3 followed the Korsmeyer-Peppas model (*R* ^2^ = 0.963 to 0.973), whereas F3, F5, and F6 followed the zero-order release (*R* ^2^ = 0.989 to 0.992) over a period of 24 hours. 

Again, the Korsmeyer-Peppas model was employed in the *in vitro* insulin permeation behavior analysis of these formulations to find out permeation mechanisms: Fickian (nonsteady) diffusional release when *n* ≤ 0.5, case-II transport (zero-order) when *n* ≥ 1, and non-Fickian, “anomalous” release when the value of n is in between 0.5 and 1 [29]. The determined values of diffusion exponent (*n*) ranged between 0.526 and 0.912 (Table 2), indicating that the drug permeation from these insulin-loaded microemulsions followed the non-Fickian, “anomalous” mechanism.

From the above results, formulation F3 (isopropyl myristate as the oil phase) and F6 (oleic acid as the oil phase) were selected for further study based on their higher permeation fluxes through the excised mouse skin than their respective microemulsions. These selected microemulsions were evaluated for further *in vitro *skin permeation using goat ear skin as higher animal skin, and the result showed higher permeation profile of microemulsion F6 than that of F3 over 24 hours. The determined permeation flux of F6 microemulsion (4.47 ± 0.09 *μ*g/cm^2^/hour) through goat skin was also higher than that of F3 microemulsion (1.64 ± 0.04 *μ*g/cm^2^/hour) (Table 1). This result coincided well with *in vitro *permeation flux data using mouse skin. The most likely mechanism for different permeation profiles for these two microemulsions may be due to directly insulin permeation from the droplets of microemulsions to the stratum corneum without microemulsion fusion to the stratum corneum and subsequent permeation enhancement.

### 3.3. Effect of DMSO as Skin Permeation Enhancer on Insulin-Loaded Microemulsions

**Figure 3:**
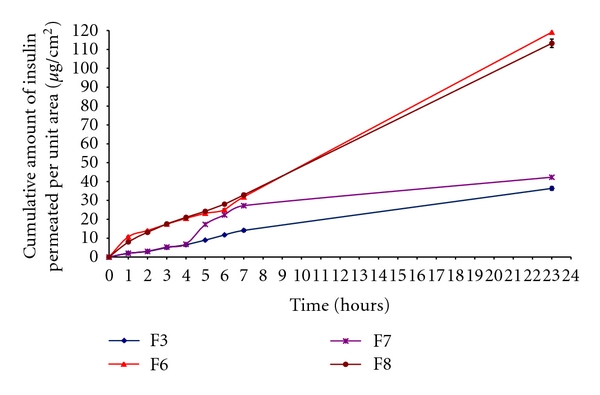
*In vitro* insulin permeation profile through goat skin per unit area from selected insulin-loaded microemulsions (F3 and F6) without and with 2% DMSO as permeation enhancer (F7 and F8). (mean ± standard error, *n* = 3).

We also investigated *in vitro* skin permeation of selected microemulsions (F3 and F6) containing insulin through goat skin using 2% dimethyl sulfoxide (DMSO) as an additional permeation enhancer for further improvement of insulin permeation. We also found higher *in vitro *permeation fluxes in case of microemulsions with permeation enhancer (2% DMSO) than those in case of microemulsions without permeation enhancer (Table 1). It was also observed that the microemulsion (F7) containing 10% oleic acid, 40% aqueous phase, and 50% surfactant phase with 2% DMSO as permeation enhancer showed maximum permeation flux (4.93 ± 0.12 *μ*g/cm^2^/hour) compared to the microemulsion containing 30% isopropyl myristate, 10% aqueous phase, and 60% surfactant phase with 2% DMSO (1.91 ± 0.05 *μ*g/cm^2^/hour). The comparative *in vitro *permeation of insulin from the selected insulin-loaded microemulsions with (F7 and F8) and without (F3 and F6) 2% DMSO as the permeation enhancer through goat skin is presented in Figure 3.

Permeation enhancers are the substances that facilitate the absorption of penetrant through the skin by temporarily diminishing the impermeability of the skin. They may act by one or more of the three main mechanisms to increase the skin permeability [26, 28, 30]: (i) improved partition of the drug or solvent into stratum corneum, (ii) disruption of highly ordered structure of stratum corneum lipid, and (iii) interaction with intracellular proteins. In the present investigation, we have used 2% DMSO as permeation enhancer. DMSO is one of the earliest and most widely studied penetration enhancers [30]. As permeation enhancer, DMSO may be operating via both mechanisms, that is, reducing the skin's resistance and aiding drug partitioning. DMSO is strongly hygroscopic and increases the hydration of the tissue [31]. It also interacts with stratum corneum protein [32], thereby, increasing the permeability of insulin through the skin. DMSO can increase the flux by reducing the resistance of both intercellular and transcellular routes. Another possible mechanism for flux enhancement may be due to delamination of horny layer by stress resulting from cross-currents of highly water interactive DMSO and water [33]. DMSO is also shown to remove substantial amounts of polar lipids at physiological temperature and this lipid extraction may be a mechanism operative in reducing the barrier function of the skin [34].

### 3.4. Droplet Size and Zeta Potential

**Figure 4:**
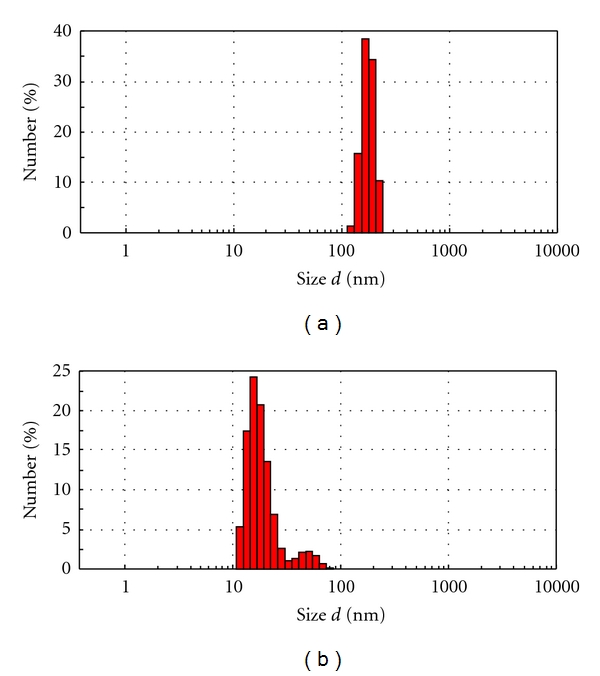
The droplet size distribution curves of the selected insulin-loaded microemulsions: (a) F3 and (b) F6.

The average droplet sizes and zeta potentials of the above selected two formulations (F3 and F6) were determined by a laser scattering particle size analyzer (MALVERN ZETASIZER, MAL500999). The average droplet sizes of F3 and F6 microemulsions were 1.0245 *μ*m and 0.415 *μ*m, respectively. The droplet size distribution curves of these two insulin-loaded microemulsions were presented in Figures 4(a) and 4(b). Due to the small droplet size of F6 microemulsion, its surface areas are assumed to be high. Therefore, droplets of microemulsion settled down to close contact with the skin providing high concentration gradient and improved insulin permeation from formulation F6 (containing lower amount of 10% oleic acid, 40% aqueous phase, and 50% surfactant phase). 

Zeta potentials of F3 and F6 microemulsions were −33.10 mV and −19.40 mV, respectively. The skin has also slight negative charge. So, the negative zeta potentials of these microemulsions containing insulin might cause little influence in improved drug permeation through skin due to electrostatic repulsion between the same charge of the skin surface and the microemulsion.

### 3.5. Stability of Microemulsions

All these insulin-loaded microemulsions were subjected to stability evaluation by centrifugation method. After 5 hours of centrifugation at 1250 rpm, these microemulsions were found to be stable as there was no sign of phase separation. 

## 4. Conclusion

The major problems of conventional insulin therapies possess several drawbacks like lower stability to different pH and enzymatic system. For large molecular peptides like insulin, it is very difficult to improve the permeation efficiency through skin after its transdermal administration. But, the microemulsion-based transdermal drug delivery systems may be a better alternative for the conventional insulin therapy. The insulin-loaded microemulsion containing 10% oleic acid, 38% aqueous phase, and 50% surfactant phase with 2% DMSO as permeation enhancer showed maximum permeation flux and can be transdermally administered in the treatment of insulin-dependent diabetes mellitus with improved patient compliance. This study highlighted the efficacy of insulin-loaded microemulsions for enhanced *in vitro *transdermal permeation through both excised mouse skin and goat skin and suggested the need for further detailed *in vivo *investigations.

## Figures and Tables

**Table 1:** Compositions of insulin-loaded microemulsions and permeation fluxes (*μ*g/cm^2^/hour) through excised mouse and goat skin.

Formulation no.	Oil phase		Surfactant and cosurfactant (3 : 1)	Water	DMSO	Permeation fluxes (*μ*g/cm^2^/hour)^†^	
	Isopropyl myristate	Oleic acid	(Tween 80 and isopropyl alcohol)			Mouse skin	Goat skin
F1	40%	—	50%	10%	—	1.19 ± 0.04	ND*
F2	35%	—	55%	10%	—	2.14 ± 0.06	ND*
F3	30%	—	60%	10%	—	2.45 ± 0.05	1.64 ± 0.04
F4	—	20%	40%	40%	—	4.48 ± 0.09	ND*
F5	—	15%	45%	40%	—	4.65 ± 0.12	ND*
F6	—	10%	50%	40%	—	5.09 ± 0.07	4.47 ± 0.09
F7	30%	—	60%	08%	2%	ND*	1.91 ± 0.05
F8	—	10%	50%	38%	2%	ND*	4.93 ± 0.12

ND*: Not determined.

^†^Mean ± Standard Error, *n* = 3.

**Table 2:** Results of curve fitting of the *in vitro *skin permeation data of various insulin-loaded microemulsions.

Formulation codes	*R* ^2^ values				Diffusional exponent (*n*)
	Zero order	First order	Higuchi	Korsmeyer-Peppas	
F1	0.926	0.591	0.886	0.973	0.426
F2	0.888	0.551	0.914	0.966	0.535
F3	0.802	0.511	0.921	0.963	0.652
F4	0.992	0.598	0.752	0.954	0.833
F5	0.993	0.595	0.741	0.961	0.841
F6	0.989	0.580	0.740	0.923	0.912

## References

[1] Peltola S, Saarinen-Savolainen P, Kiesvaara J, Suhonen TM, Urtti A (2003). Microemulsions for topical delivery of estradiol. *International Journal of Pharmaceutics*.

[2] Yuel Y, San-ming L, Li-min Y, Pan D, Da-fangl Z (2007). Physicochemical properties and evaluation of microemulsion systems for transdermal delivery of meloxicam. *Chemical Research in Chinese Universities*.

[3] Gasco MR (1997). Microemulsions in the pharmaceutical field: perspectives and applications. *Industrial Applications of Microemulsions*.

[4] Sintov AC, Botner S (2006). Transdermal drug delivery using microemulsion and aqueous systems: influence of skin storage conditions on the in vitro permeability of diclofenac from aqueous vehicle systems. *International Journal of Pharmaceutics*.

[5] El Maghraby GM (2010). Self-microemulsifying and microemulsion systems for transdermal delivery of indomethacin: effect of phase transition. *Colloids and Surfaces*.

[6] Baroli B, López-Quintela MA, Delgado-Charro MB, Fadda AM, Blanco-Méndez J (2000). Microemulsions for topical delivery of 8-methoxsalen. *Journal of Controlled Release*.

[7] Delgado-Charro MB, Iglesias-Vilas G, Blanco-Méndez J, López-Quintela MA, Marty JP, Guy RH (1997). Delivery of a hydrophilic solute through the skin from novel microemulsion systems. *European Journal of Pharmaceutics and Biopharmaceutics*.

[8] Kreilgaard M (2002). Influence of microemulsions on cutaneous drug delivery. *Advanced Drug Delivery Reviews*.

[9] El Maghraby GM (2008). Transdermal delivery of hydrocortisone from eucalyptus oil microemulsion: effects of cosurfactants. *International Journal of Pharmaceutics*.

[10] Hermeling S, Crommelin DJA, Schellekens H, Jiskoot W (2004). Structure-immunogenicity relationships of therapeutic proteins. *Pharmaceutical Research*.

[11] Nayak AK (2010). Advances in therapeutic protein production and delivery. *International Journal of Pharmacy and Pharmaceutical Sciences*.

[12] Tripathi KD (1999). Insulin, oral hypoglycaemics and glucagon. *Essentials of Medical Pharmacology*.

[13] Davis SN, Brunton LL, Lazo JS, Parker KL (2005). Insulin, oral hypoglycemic agents, and the pharmacology of endocrine pancreas. *Goodman and Gilman's the Pharmacological Basis of Therapeutics*.

[14] Shah D, Agarawal V, Parikh R (2010). Non invasive insulin delivery system: a review. *International Journal of Applied Pharmaceutics*.

[15] Khafagy ES, Morishita M, Onuki Y, Takayama K (2007). Current challenges in non-invasive insulin delivery systems: a comparative review. *Advanced Drug Delivery Reviews*.

[16] Kennedy FP (1991). Recent developments in insulin delivery techniques: current status and future potential. *Drugs*.

[17] Sen A, Daly ME, Hui SW (2002). Transdermal insulin delivery using lipid enhanced electroporation. *Biochimica et Biophysica Acta*.

[18] Pillai O, Nair V, Panchagnula R (2004). Transdermal iontophoresis of insulin: IV. Influence of chemical enhancers. *International Journal of Pharmaceutics*.

[19] Pillai O, Nair V, Sivaprasad N, Panchagnula R (2003). Transdermal iontophoresis of insulin: II. Physicochemical considerations. *International Journal of Pharmaceutics*.

[20] Amnon CS, Wormser U (2007). Topical iodine facilitates transdermal delivery of insulin. *Journal of Controlled Release*.

[21] Cevc G (2003). Transdermal drug delivery of insulin with ultradeformable carriers. *Clinical Pharmacokinetics*.

[22] King MJ, Badea I, Solomon J, Kumar P, Gaspar KJ, Foldvari M (2002). Transdermal delivery of insulin from a novel biphasic lipid system in diabetic rats. *Diabetes Technology and Therapeutics*.

[23] Yang JH, Kim YI, Kim KM (2002). Preparation and evaluation of aceclofenac microemulsion for transdermal delivery system. *Archives of Pharmacal Research*.

[24] Derle DV, Sagar BSH, Sagar P (2006). Microemulsion as a vehicle for transdermal permeation of nimesulide. *Indian Journal of Pharmaceutical Sciences*.

[25] Pillai O, Panchagnula R (2004). Transdermal iontophoresis of insulin: VI. Influence of pretreatment with fatty acids on permeation across rat skin. *Skin Pharmacology and Physiology*.

[26] Barry BW (1983). *Dermatological Formulations: Percutaneous Absorption*.

[27] Gannu R, Vishnu Yamsani V, Rao Yamsani M (2008). Enhancement potential of *Aloe vera* on permeation of drugs with diverse lipophilicities across rat abdominal skin. *Current Trends in Biotechnology and Pharmacy*.

[28] Nayak AK, Mohanty B, Sen KK (2010). Comparative evaluation of in vitro diclofenac sodium permeability across excised mouse skin from different common pharmaceutical vehicles. *International Journal of PharmTech Research*.

[29] Patel VM, Prajapati BG, Patel MM (2007). Effect of hydrophilic polymers on buccoadhesive Eudragit patches of propranolol hydrochloride using factorial design. *AAPS PharmSciTech*.

[30] Pathan IB, Setty CM (2009). Chemical penetration enhancers for transdermal drug delivery systems. *Tropical Journal of Pharmaceutical Research*.

[31] Idson B (1978). Hydration and percutaneous absorption. *Current Problems in Dermatology*.

[32] Barry BW (1987). Mode of action of penetration enhancers in human skin. *Journal of Controlled Release*.

[33] Chandrasekaran SK, Champbell PS, Michels AS (1977). Effects of dimethylsulfoxide on drug permeation through human skin. *AIChE Journal*.

[34] Embery G, Dugard PH (1971). The isolation of dimethylsulphoxide soluble components from human epidermal preparation: a possible mechanism of action of dimethylsulphoxide in affecting percutaneous migration phenomena. *Journal of Investigative Dermatology*.

